# Surgical Treatment of Dorsal Carpal Ganglions: A Retrospective Clinical Trial

**DOI:** 10.7759/cureus.10252

**Published:** 2020-09-05

**Authors:** Nazmi Bülent ALP, Gokhan Akdag

**Affiliations:** 1 Orthopaedics and Traumatology, Bursa City Training and Research Hospital, Bursa, TUR; 2 Orthopaedics and Traumatology, Istanbul Bahcelievler State Hospital, Istanbul, TUR

**Keywords:** wrist, dorsal carpal ganglion, recurrence, ganglionectomy, scapholunate ligament

## Abstract

Objectives

Our primary goal in this study was to investigate whether the surgical treatment we performed on the patients with dorsal carpal ganglion was effective rather than evaluating preoperative and postoperative functional results.

Methods

The retrospective study included patients who were operated with open technique due to dorsal wrist ganglion at a single center between March 1, 2015, and December 1, 2017, and were followed for at least six months. Thirty-three wrists of 32 patients (31 unilateral and 1 bilateral) were operated. During follow-ups, complication rates, patient satisfaction, and recurrence rates were evaluated.

Results

Of the 32 patients, 19 were females and 13 were males. Mean age of the patients was 38.6 ± 13.0 years (min-max = 19-60 years). Excision was performed on 28 right and 5 left wrists. The follow-up period of patients varied between 6 months and 38 months (mean = 21.7 ± 9.4 months). Recurrence was detected in four (12.5%) patients during the postoperative period. Complex regional pain syndrome occurred in two (6.25%) patients. Joint stiffness developed in six (18.75%) patients during the postoperative period. When recurrent cases were excluded from our cases, the satisfaction rate was 87.5%.

Conclusions

Open surgical excision has satisfactory results that cannot be achieved with conservative treatment in the treatment of symptomatic dorsal ganglia. In order to keep the recurrence rate at the minimum level after surgery, it is critical to excise the ganglion and pedicle without leaving any residual tissue. Proper surgical technique improves patient satisfaction by eliminating pain and cosmetic discomfort.

## Introduction

Ganglia, which have been known since the time of Hippocrates, come from the term “ganglion” in Greek, which terminologically means “tissue knot”, and is the most common soft tissue tumor of the hand and wrist [1]. Ganglion is filled with a viscous liquid consisting of hyaluronic acid, globulin, albumin, and glucosamine [2]. The vast majority of patients admit to the orthopedics or hand surgery outpatient clinics with a mass that is palpated in the dorsum of the wrist, cosmetically unwanted, causing pain and limitation in joint movements of the wrist.

Dorsal carpal ganglia (DCGs) traditionally originate from where the dorsal part of the scapholunate ligament (SL) meets the joint capsule. The pain is a result of the compression on the posterior interosseous nerve terminal branches situated between the third and fourth extensor compartments. Preoperative diagnosis can be confirmed by transillumination or needle aspiration performed on DCG, as well as with the imaging methods such as ultrasound and MRI. There is a vast amount of studies in the literature explaining its etiopathogenesis. Trauma, mucoid degeneration, and one-way valve mechanism causing ligament injury are the most accepted factors in the etiopathogenesis [3-5]. Despite high recurrence rate, needle aspiration, cortisone injection, and splinting are performed conservatively in patients who do not want to undergo surgery. Surgical treatment options are open surgery or arthroscopic resection of ganglia, which was defined by Osterman and Raphael [6].

Our primary goal in this study was to investigate whether the surgical treatment we applied is effective rather than evaluating the pre- and postoperative functional results.

## Materials and methods

**Figure 1:**
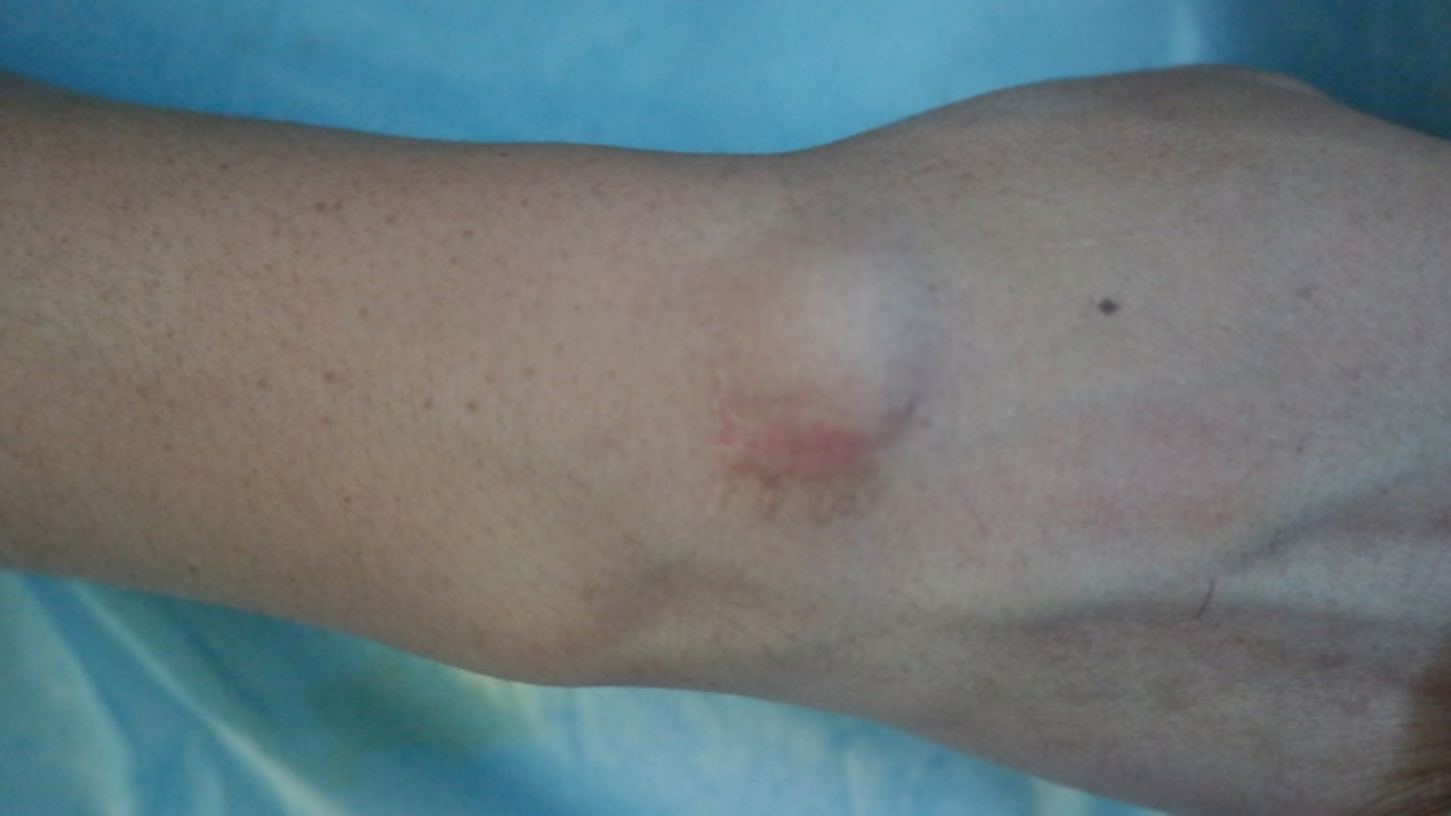
Preoperative appearance of the classic dorsal carpal ganglion on the wrist before surgery.

This retrospective study was approved by the Local Ethics Committee. Informed consent form was obtained from all patients. Patients who underwent dorsal wrist carpal ganglion surgery by open technique between March 1, 2015, and December 1, 2017, were included in the study. Patients under the age of 18, those who were operated arthroscopically (two patients), and those without adequate medical records and with a follow-up less than six months were excluded from the study. Thirty-three wrists of 32 patients (31 unilateral and 1 bilateral) were operated. Excision was performed on 28 right and 5 left wrists. Of the 32 patients, 19 were females and 13 were males. Mean age of the patients was 38.6 ± 13.0 years (min-max = 19-60 years). The follow-up period of patients varied between 6 and 38 months (mean = 21.7 ± 9.4 months). Demographical and clinical data of the patients are presented in Table 1. All of the patients admitted to the clinic with a mass that was aesthetically disturbing and limiting wrist movements (Figure 1). Of the 32 patients, 9 and 23 patients stated that they experience more pain during hyperflexion and hyperextension, respectively. One of the patients underwent the surgery due to recurrence, whereas the others underwent primary surgeries. Images of the wrists of all patients were obtained in two directions preoperatively, and due to cost-effectiveness, ultrasonography was requested from all patients instead of MRI.

**Table 1:** Demographical and clinical data of the patients (n = 32).

Gender	n (%)
Male, %	13 (40.6)
Female, %	19 (59.4)
Age (years), mean ± SD (min-max)	38.6 ± 13.0 (19-60)
Surgical site	n (%)
Left	5 (15.2)
Right	28 (84.8)
Unilateral	31 (96.9)
Bilateral	1 (3.1)
Complication, n (%)	12 (37.5)
Recurrence, n (%)	4 (12.5)
Mean follow-up (months), mean ± SD (min-max)	21.7 ± 9.4 (6-38)

Surgical technique

**Figure 2:**
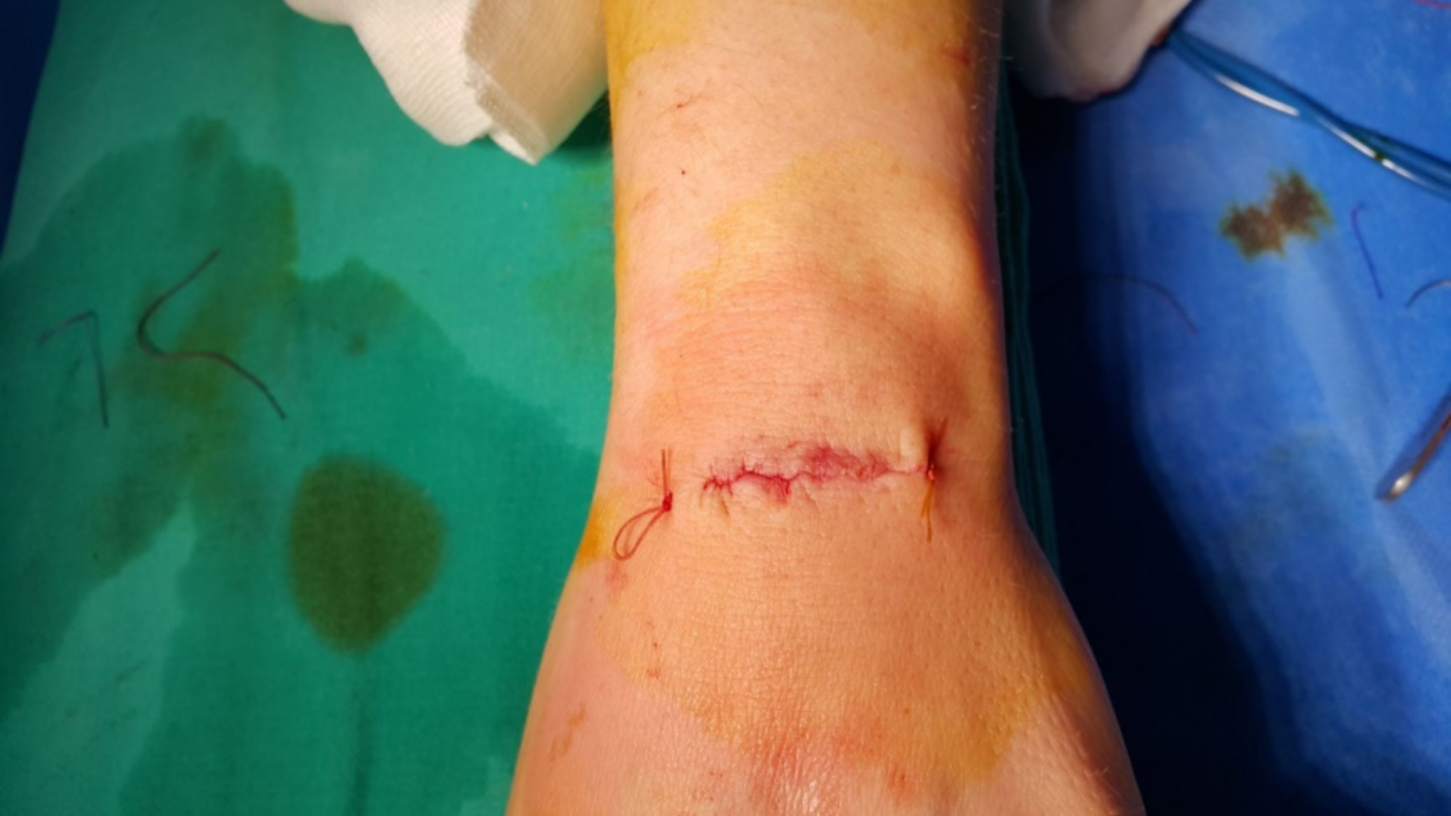
Transverse incision for dorsal carpal ganglionectomy.

**Figure 3:**
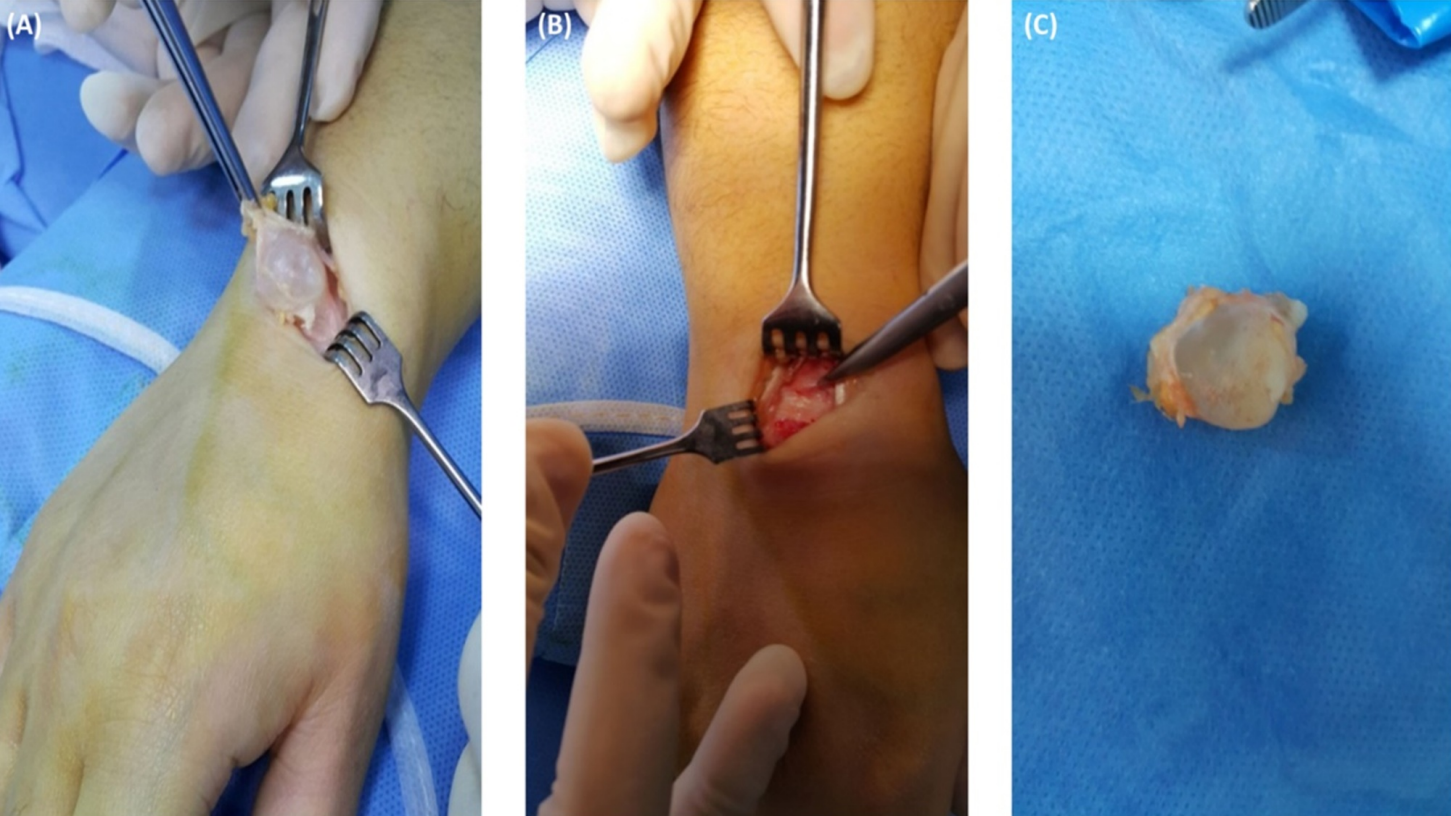
Total excision of the intact ganglion with pedicle.

All patients whose routine surgery preparations were completed were operated under general or regional anesthesia by using a pneumatic tourniquet. Surgical opening was achieved with a 2-3 cm transverse incision over the DCG (Figure 2). Since the DCGs are generally located between the extensor pollicis longus and extensor digitorum communis tendons, these two tendons are preserved, and the ganglion and pedicle are released (Figure 3A). Ganglion was dissected through SL ligament together with the adjacent joint capsule (Figure 3B). Ganglion, pedicle, and capsular extensions were excised off with a portion of the SL ligament (Figure 3C). Hemostasis was achieved by releasing the pneumatic tourniquet. We did not close the joint capsule in any patient since it caused limitation during wrist movements. In all cases, the skin incision was sutured subcutaneously. All patients were discharged on the day of the operation with a short arm splint. At the first control one week later, immobilization of the wrist was terminated. Strengthening movements were avoided for the first four to six weeks.

The medical records of patients who were operated by a single surgeon by performing the aforementioned surgical method and underwent the same rehabilitation procedures were reviewed retrospectively in this study, and whether the patients benefited in the operation was questioned and results were discussed by using the data in the literature.

## Results

**Figure 4:**
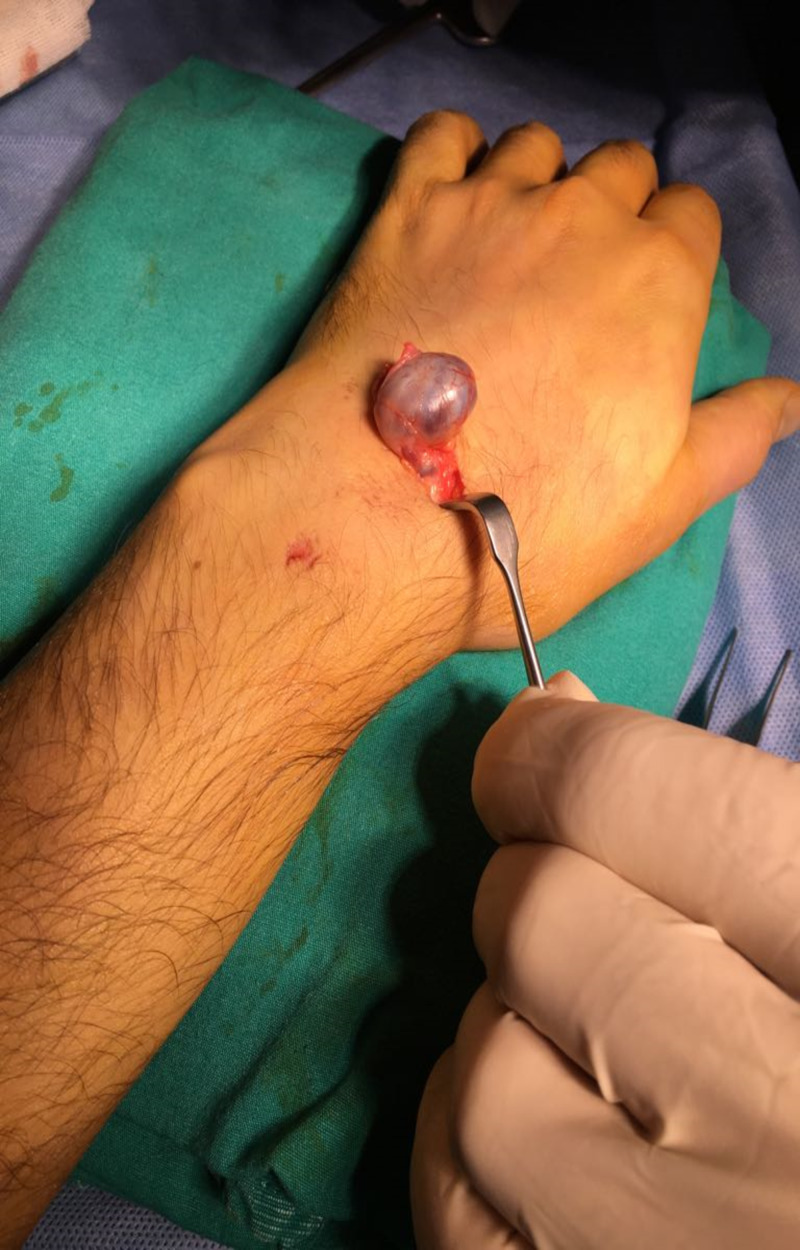
Intralesional hemorrhage in the ganglion.

During DCG excision, ganglion pedicle, adjacent joint capsule, and a small portion of SL were excised in all cases. All samples sent to the pathology were reported in accordance with the classical pathology of DCG. Macroscopically, intralesional hemorrhage was detected in one ganglion due to possible recurrent trauma (Figure 4). Recurrence was detected in four (12.5%) patients in the postoperative period. Fluid accumulation was confirmed by control ultrasonographic examination. While one of the patients with recurrence agreed to be re-operated, the other three did not want the same surgical intervention again and thus aspiration was performed and methylprednisolone was administered in these patients by needle. Complex regional pain syndrome developed in two (6.25%) patients. Joint stiffness developed in six (18.75%) patients in the postoperative period. After physical therapy varying between six and eight weeks, the range of motion was regained. No neuroma formation was observed. No superficial infection, hematoma formation, or keloid formation was observed at the wound site. During the follow-up period, scapholunate instability was not detected in any patient. There was no tendon injury or any neurovascular injury in any of the operated patients. When cases with recurrence were excluded from our cases, the satisfaction rate was 87.5%.

## Discussion

Although DCGs are benign in nature and are frequently encountered in orthopedics hand surgery practice, they restrict wrist joint movements, causing loss of function, pain, and poor cosmetic appearance. Although the DCGs are tried to be treated by performing conservative methods such as aspiration, aspiration + steroid injection, aspiration + hyaluronidase, and aspiration + splinting, the recurrence rates were reported between 15% and 77% in the literature [7-13]. Due to high recurrence rates, surgical treatment has become the gold standard in patients who are symptomatic. In particular, the work by Angelides and Wallace has become a guide by reducing recurrence rates down to 1-2% [14]. These researchers reported in their study that the dorsal ganglion pedicle penetrates the wrist capsule and showed macroscopically that there is a folded channel associated with the scapholunate joint. The researchers' suggestion is the excision of the ganglion, pedicle of the ganglion, adjacent joint capsule, and a small portion of the SL ligament in which the pedicle penetrates. This is because in some cases, the residual channel will act as a one-way valve mechanism and can create recurrence by pumping fluid from the wrist joint to the ganglion. The operation technique we applied in our study was to excise the ganglion, its associated pedicle, and a portion of SL ligament, as suggested by Angelis and Wallace. The low recurrence rate that was in parallel with the literature in our study reveals the importance of the described surgical technique. Besides the recurrence, another important complication observed after ganglionectomy is joint stiffness. Rizzo et al. [15] reported restriction in the wrist movement after surgery in 25% of dorsal ganglion patients that they followed for a minimum of two years after arthroscopic resection. Mathoulin and Gras [16] stated that arthroscopic ganglion excision caused less movement restriction compared to open surgery. In our case series, movement limitation was detected in the wrist joint in six (18.75%) of our patients who operated with open surgery techniques. We have stated that we did not suture the joint capsule to prevent limitations. Nevertheless, blunt dissection performed during the release of the ganglion causes adhesions in an anatomical region where there are many ligamentous structures such as wrist, leading to joint stiffness during the recovery period. The most important reason for the operation indications of this group of patients with a ganglion in the dorsal part of the wrist is pain. Gude and Morelli [17] reported that compression of the posterior interosseous nerve was the cause of pain around the ganglion that was disturbing the patient. Dias et al. [18] reported that pain present in the preoperative period was a risk factor for postoperative ongoing pain. Pain complaints were present in our patients, especially those with limited wrist motion. After the six to eight weeks of rehabilitation program, pain complaints disappeared with gaining wrist movements. When we considered all the patients, no residual pain was observed in the incision site or around during the follow-up period. In our study, no scapholunate instability in any of our patients was encountered during follow-up. However, there are publications in the literature indicating scapholunate instability after DCG excision [19-21]. In these publications, it is stated that the SL ligament is weakened while it is excised, and this causes SL instability. On the other hand, trauma weakness in the SL has been reported to be one of the etiological causes of the dorsal ganglion [6,7,13]. In the light of literature knowledge, there is a relationship between SL instability and dorsal ganglia, but in this study with open surgical technique, no data related to instability were obtained.

There are missing parts of the retrospective study. The relatively low number of cases is a limitation. A study conducted on a larger patient group will allow more inclusive data to be collected. The inclusion of functional results could make the study more efficient, but functional measurements could not be tolerated by the patients due to the fact that the measurements should have been conducted between examinations in an ordinary public hospital with a limited number of outpatient polyclinics. Since the purpose of our study was to show whether the patients benefited from the surgical treatment we performed, we think that the aforementioned limitation can be ignored.

## Conclusions

The results of dorsal ganglion excisions made with open surgical technique are satisfying. In order to keep the recurrence rate low, the most crucial point of the operation is to remove the pedicle together with a small portion of the associated SL ligament with meticulous blunt dissection without leaving the residue of the pedicle.

## References

[1] Soren A (1966). Pathogenesis and treatment of ganglion. Clin Orthop Relat Res.

[2] Slutsky DJ (2016). Techniques in Wrist and Hand Arthroscopy. 2nd edn.

[3] Naam NH (2012). Synovial fistula as a complication of recurrent dorsal wrist ganglion excision: case report. J Hand Surg Am.

[4] Nishikawa S, Toh S, Miura H, Arai K, Irie T (2001). Arthroscopic diagnosis and treatment of dorsal wrist ganglion. J Hand Surg Br.

[5] Andrén L, Eiken O (1971). Arthrographic studies of wrist ganglions. J Bone Joint Surg Am.

[6] Osterman AL, Raphael J (1995). Arthroscopic resection of dorsal ganglion of the wrist. Hand Clin.

[7] Breidahl WH, Adler RS (1996). Ultrasound-guided injection of ganglia with coricosteroids. Skeletal Radiol.

[8] Zubowicz VN, Ishii CH (1987). Management of ganglion cysts of the hand by simple aspiration. J Hand Surg Am.

[9] Korman J, Pearl R, Hentz VR (1992). Efficacy of immobilization following aspiration of carpal and digital ganglions. J Hand Surg Am.

[10] Varley GW, Needoff M, Davis TR, Clay NR (1997). Conservative management of wrist ganglia: aspiration versus steroid infiltration. J Hand Surg Br.

[11] Paul AS, Sochart DH (1997). Improving the results of ganglion aspiration by the use of hyaluronidase. J Hand Surg Br.

[12] Jagers Op Akkerhuis M, Van Der Heijden M, Brink PR (2002). Hyaluronidase versus surgical excision of ganglia: a prospective, randomized clinical trial. J Hand Surg Br.

[13] Richman JA, Gelberman RH, Engber WD, Salamon PB, Bean DJ (1987). Ganglions of the wrist and digits: results of treatment by aspiration and cyst wall puncture. J Hand Surg Am.

[14] Angelides AC, Wallace PF (1976). The dorsal ganglion of the wrist: its pathogenesis, gross and microscopic anatomy, and surgical treatment. J Hand Surg Am.

[15] Rizzo M, Berger RA, Steinmann SP, Bishop AT (2004). Arthroscopic resection in the management of dorsal wrist ganglions: results with a minimum 2-year follow-up period. J Hand Surg Am.

[16] Mathoulin C, Gras M (2017). Arthroscopic management of dorsal and volar wrist ganglion. Hand Clin.

[17] Gude W, Morelli V (2008). Ganglion cysts of the wrist: pathophysiology, clinical picture, and management. Curr Rev Musculoskelet Med.

[18] Dias JJ, Dhukaram V, Kumar P (2007). The natural history of untreated dorsal wrist ganglia and patient reported outcome 6 years after intervention. J Hand Surg Eur Vol.

[19] Duncan KH, Lewis RC, Jr Jr (1988). Scapholunate instability following ganglion cyst excision. A case report. Clin Orthop Relat Res.

[20] Crawford GP, Taleisnik J (1983). Rotatory subluxation of the scaphoid after excision of dorsal carpal ganglion and wrist manipulation--a case report. J Hand Surg Am.

[21] Clay NR, Clement DA (1988). The treatment of dorsal wrist ganglia by radical excision. J Hand Surg Br.

